# Coffee and Caffeine Consumption for Human Health

**DOI:** 10.3390/nu13092918

**Published:** 2021-08-24

**Authors:** Raquel Abalo

**Affiliations:** 1 Department of Basic Health Sciences, Faculty of Health Sciences, University Rey Juan Carlos (URJC), 28922 Alcorcón, Spain; raquel.abalo@urjc.es; Tel.: +34-91-488-8854; 2 High Performance Research Group in Physiopathology and Pharmacology of the Digestive System (NeuGut-URJC), URJC, 28922 Alcorcón, Spain; 3 Associated I+D+i Unit to the Institute of Medicinal Chemistry (IQM), Scientific Research Superior Council (CSIC), 28006 Madrid, Spain; 4 Working Group of Basic Sciences in Pain and Analgesia of the Spanish Pain Society (Grupo de Trabajo de Ciencias Básicas en Dolor y Analgesia de la Sociedad Española del Dolor), 28046 Madrid, Spain

Coffee is one of the most popular and consumed beverages worldwide, and caffeine is its best-known component, present also in many other beverages (tea, soft drinks, energy drinks), foodstuffs (cocoa, chocolate, guarana), sport supplements and even medicines. Besides caffeine, many other components, either beneficial for health (chlorogenic acids, polyphenols, diterpenes, micronutrients, melanoidins, fiber) or not (lipids in unfiltered coffee, or acrylamide resulting from coffee bean roasting), are present in coffee. Just to illustrate the scientific interest of coffee and caffeine, when these terms are combined in PubMed (as “coffee OR caffeine”), almost 50,000 papers can be retrieved (as of 10 August 2021). Furthermore, from 2000 to 2020, the number of manuscripts published per year has more than doubled (from 972 to 2601). Within this context of increasing interest in the topic, the Special Issue (SI) on “Coffee and Caffeine Consumption for Human Health” has collected twenty-one manuscripts (five narrative reviews and sixteen original articles, including two meta-analyses).

Most of the original reports obtained information on coffee or caffeine consumption in humans through dietary surveys or interviews. These studies have the limitation of recall bias. In addition, caffeine content needs to be estimated for most foods from packaging, databases, scientific literature or extrapolated from similar foods, although in some cases it was directly measured from samples of coffee or soft drinks. Rochat et al. found that, in agreement with reports from other high-income countries, coffee is the main source of caffeine consumption in Switzerland, mostly consumed early in the morning (6–9 am), although some differences were found across age groups, smoking status, and linguistic regions [1]. Cultural differences in coffee/caffeine consumption are important and may contribute to the health effects observed in different geographic regions. Furthermore, coffee/caffeine consumption may be likewise modulated by expectation (placebo) effects and vice versa. Mendes et al. translated, adapted, and validated the Caffeine Expectancy Questionnaire (CaffEQ), originally designed for the American population [2], to the Brazilian culture (CaffEQ-BR), and confirmed that coffee is the main source of daily caffeine intake in Brazil [3], the largest coffee producer and exporter in the world market.

Most studies tried to determine whether there is an association between coffee/caffeine intake and different health outcomes. Rodas et al. specifically evaluated the effect of caffeine intake, physical activity levels, and sedentary behavior on the inflammatory status in healthy staff and students at the University of the Balearic Islands (Mallorca, Spain). In this sample, sedentary behavior and body fat accumulation had clear pro-inflammatory effects, whereas regular but relatively low caffeine consumption (whose main source was also coffee) could not be demonstrated to exert robust anti-inflammatory effects [4]. Antwerpes et al., analyzed the relationship between regular coffee intake and neurocognitive performance in patients coinfected with human immunodeficiency virus and hepatitis C virus, who experience an accelerated aging process and cognitive impairment. The authors showed a positive association between elevated coffee intake (three or more cups per day) and neurocognitive functioning parameters, even after adjusting for liver disease correlates, suggesting that coffee intake may be neuroprotective in these patients [5]. Likewise, Herden and Weissert studied the effect of coffee and caffeine consumption on patients with multiple sclerosis (MS)-related fatigue. Importantly, the authors showed that coffee intake did not cause severe side effects in MS patients and identified a specific set of patients who might benefit from coffee consumption [6]. One meta-analysis evaluated the effect of caffeine consumption on the risk and progression of Parkinson’s disease (PD). Individuals consuming caffeine on a regular basis had a significantly lower risk of developing PD during follow-up evaluation, and those that already had the disease and consumed caffeine showed a significantly decelerated PD progression. However, Hong et al. could not determine the optimal daily dosage or food source of caffeine [7]. According to the review by Jee et al., coffee/caffeine neuroprotective effects seem to be broader and sex- and age-specific. Indeed, they concluded that caffeine consumption reduces the risk of stroke, dementia, and depression in women and that of PD in men. Nevertheless, it may increase sleep disorders and anxiety disorders in adolescence in both men and women. They suggested that caffeine use should be individualized according to sex (and age) in the context of neurologic and psychiatric diseases [8]. Nowaczewska et al., on the other hand, reviewed the ambiguous role of caffeine in migraine headache. They did not find any scientific evidence showing that a single dose of caffeine may trigger migraine, although it may influence migraines (i.e., through its vasoconstrictor actions during the premonitory symptoms). Chronic caffeine overuse may lead to migraine chronification and sudden caffeine cessation may trigger migraine attacks. Thus, as recommended by the authors, migraine sufferers should avoid caffeine withdrawal headache by keeping a consistent daily intake, not exceeding 200 mg [9].

Three studies evaluated the relationship of coffee consumption with the risk of metabolic, endocrine, or cardiovascular diseases according to genetic polymorphisms. Using data from the Korean Genome and Epidemiology Study (KGES), Jin et al. identified five single nucleotide polymorphisms (SNPs) related with habitual coffee consumption in this Korean population and showed the lowest risk of prediabetes and type 2 diabetes among black-coffee consumers with minor alleles of these SNPs compared with those with major alleles [10]. Using data from KGES too, Han et al. found that subsets of genetic variants in the adenosine receptors (involved in caffeine signaling) gene family modulate the effect of coffee intake on dyslipidemia risk in a sex-dependent manner [11]. In the third study, Liu et al. found that consumption of coffee was significantly associated with a decreased risk of coronary heart disease among Taiwanese adults carrying the GG genotype of TRIB1 (tribbles pseudokinase 1, a gene involved in cholesterol metabolism and atherosclerosis process) [12]. These three examples of genetic studies strongly suggest that dietary guidelines for coffee intake in the prevention and management of metabolic, endocrine, and cardiovascular disorders should consider the influence of genetic polymorphisms.

The main problem with the survey/interview-based studies is the lack of accurate information regarding type (roasting) or brand of coffee, caffeine content (caffeinated, decaffeinated), methods of preparation (boiled, filtered, brewed), and consumption of other caffeine sources. Thus, quantification of daily consumption of caffeine (and other compounds) is a real challenge in this kind of studies. Moua et al. tried to overcome this limitation by using volume of coffee consumed (not number of cups) in a dose-response meta-analysis of the association between coffee consumption and c-reactive protein, a general biomarker of chronic inflammation. Unfortunately, heterogeneity of study populations (differences in sample size; cultural differences in coffee composition; relevant individual confounders such as age, sex, body mass index, smoking, alcohol intake, diet, activity, comorbidities, etc.) produced inconsistent associations [13]. Thus, in addition to collecting detailed information on coffee type and preparation method, measuring biomarkers of coffee consumption such as urinary metabolites may be helpful to more precisely determine the amounts of bioactive compounds consumed and their effects. In this sense, Wu and Chen explored the association between urine caffeine metabolites and urine flow rate, using data from the US National Health and Nutrition Examination Survey (NHANES) and metabolomics for urine analyses. The association was positive, with more metabolites showing certain flow-dependency in males compared to females and in young compared to elderly participants [14]. This factor is important to correctly interpret urinary data regarding caffeine.

Intervention studies allow to establish more robust cause-effect associations. In this SI, two original studies used a double-blind, placebo-controlled crossover design to evaluate the ergogenic effects of caffeine. In 13 males and 17 females, Fuller et al. examined the effects of trait (long-standing pre-disposition) mental and physical energy and fatigue to changes in moods, cognitive and fine-motor task performance. The results suggest that evaluating trait may be a practical, low-cost method to control for interindividual differences in the ergogenic neurocognitive effects of caffeine, without the need for genetic testing [15]. On the other hand, Wilk et al. demonstrated that a single moderate dose of caffeine (3–6 mg/kg b.m.) increased mean power output and mean bar velocity during an explosive bench press throw in 12 male athletes habituated to caffeine ingestion, meaning that caffeine enhances performance in this context, although the long-term training effects with caffeine need to be determined [16].

This SI includes three original studies showing new data in animal models using different beverages for different purposes, somehow representative of those also addressed in humans. Ahmad et al. performed a classical toxicity study in rats of the beverage Tongkat ali, widely used in Malaysia, made of coffee infused with the additive *Eurycoma longifolia*. This study demonstrated a good safety profile for this beverage in male and female rats [17]. The other rat study, by Velázquez et al., investigated the effects of caffeine alone or as part of a green coffee extract (GCE) in lean female rats with diet-induced hepatic steatosis, as a preclinical model of non-alcoholic fatty liver disease, a highly prevalent condition nowadays, without specific pharmacological treatment. Using different techniques, including lipidomics on liver tissue, the GCE, but not caffeine alone, was found to reduce liver triglyceride levels, through a combination of different molecular mechanisms of action [18]. Whether longer treatment duration and/or higher doses might be even more effective is unknown. In the last preclinical study, a controlled laboratory trial performed in piglets, Treml et al. showed that a high dose of Red Bull, a popular energy drink among athletes containing caffeine, taurine and glucose among other compounds, increased heart rate at near sea level. However, a high dose of this beverage did not worsen tachycardia during acute short-term hypoxia (simulating high altitude conditions). The authors demonstrated that this beverage significantly increased pulmonary shunt fraction without changing distribution of pulmonary blood flow during hypoxia [19]. The specific contributions of the different components of this beverage remain to be identified.

Ruta and Farcasanu reviewed the studies evaluating the molecular mechanisms of action of caffeine in *Saccharomyces cerevisiae* as a simple model of eukaryotic cell. In addition to its three well-known mechanisms, namely intracellular mobilization of calcium, inhibition of phosphodiesterases and antagonism of adenosine receptors, the studies performed in this yeast model have confirmed that the pleiotropic effects of caffeine involve also key molecular mechanisms related with DNA repair mechanisms, cancer, and aging [20]. In contrast, Kolb et al. reviewed the mechanisms that might contribute to explain the beneficial effects of habitual coffee consumption on health. The authors excluded caffeine content as well as radical scavenging properties, anti-inflammatory activity, and genetic polymorphisms as major contributors to coffee healthy effects. Instead, they propose that the mechanisms involve a combination of factors promoting cell protection, namely upregulation of proteins with antioxidant, detoxifying and repair functions through coffee phenolic phytochemicals, as well as modulation of the gut-microbiota, through the non-digestible components of coffee (prebiotics), although this has been scarcely explored [21]. Since the gastrointestinal tract is the first body system that gets in contact with ingested coffee, Iriondo-DeHond et al. reviewed the effects produced by coffee and its components on the different constituents of the gut wall (mucosa, muscle layers, enteric nervous system), the different gastrointestinal organs, the gastrointestinal tract as a whole and the brain-gut axis, only to find that the effects of coffee and its derivatives on the health of this axis (that affect not only gastrointestinal motility, permeability and sensitivity but also a complete spectrum of central nervous functions and disorders, from emotions to neurodegeneration) have not been deeply investigated yet [22].

Altogether, the current view is that coffee/caffeine intake exerts multiple health benefits in humans, at least in specific populations (with a particular genetic profile or suffering from specific diseases), but the specific effects in the different organs and systems, as well as the mechanisms involved are far from clear. Furthermore, within the current context aiming to sustainable development, the coffee plant *Coffee* sp. and its so-far relatively neglected by-products are expected to become soon a source of ingredients for new functional foods whose properties will need to be precisely determined. We hope the readers of this SI will find inspiration for new studies on the topic.
